# A New Rubber Mask Specially Designed for General Anesthesia with Ethyl-Chloride Ampoules

**Published:** 1919-01

**Authors:** 


***A New Rubber Mask Specially Designed for General Anesthesia with Ethyl-Chloride Ampoules.*** By F. C. Barlow. *The Lancet*, September 14, 1918.

An ingenious apparatus for general anesthesia with exact dosage of ethyl-chloride, as gauged for each particular case, has been devised. It is so constructed that the whole of the ethyl-chloride contained in each graded glass ampoule is administered gradually, fully, and without loss. The whole apparatus consists of one piece of rubber with an adjustment of two tubes running at right angles. One limb of the T receives the ampoule; the other is armed in the center of its blind end with a pin projecting into the center of the lumen. This limb receives the umbilicated end of the glass ampoule of ethyl-chloride. When the ampoule is pressed home the pin first impinges on and then perforates the umbilicated end; the ethyl-chloride vapor streams out along the short, wide tube onto a gauze pad, suitably placed. The vapor diffuses throughout the pad from which it is breathed by the patient.

If before introduction the glass ampoule is slightly moistened, no difficulty will be experienced in pushing it smoothly into the tube. If one ampoule proves insufficient, it can be supplemented by another of any desired capacity.

This mask fits quite confortably. The makers are the Anglo-French Drug Company, Ltd., Holborn, London.

